# Effectiveness of foot skin protection technology in elderly patients with diabetic peripheral neuropathy

**DOI:** 10.3389/fendo.2024.1411657

**Published:** 2024-08-19

**Authors:** Lin Zhou, Pin-Fang Song, Wen Qin, Qing Jia, Fei Miao, Jiao-Jiao Bai

**Affiliations:** ^1^ Department of Endocrine Ward, Huadong Hospital Affiliated to Fudan University, Shanghai, China; ^2^ Department of Nursing, Huadong Hospital Affiliated to Fudan University, Shanghai, China; ^3^ Department of Geriatrics, Affiliated with Huadong Hospital of Fudan University, Shanghai, China; ^4^ Department of Dermatology, Affiliated Huadong Hospital, Fudan University, Shanghai, China

**Keywords:** diabetic foot, diabetic peripheral neuropathy, elderly diabetes, nursing, skin protection

## Abstract

**Objective:**

The aim of this study is to assess the effectiveness of foot skin protection technology in elderly patients with diabetic peripheral neuropathy.

**Methods:**

The foot skin protection technology was developed based on a comprehensive literature review and preliminary research conducted by our research team. Subsequently, 88 elderly patients with diabetic peripheral neuropathy and experiencing foot skin problems were recruited from two community health service centers in Shanghai. Using a random number table, the participants were randomly assigned to either the control group or the experimental group. Patients in the experimental group received foot skin protection technology interventions, while those in the control group received standard community nursing guidance for a duration of 3 months. The incidence, severity, and discomfort associated with foot skin problems were evaluated before and after the intervention period in both groups.

**Results:**

The incidence, severity, and discomfort of foot skin problems notably reduced in the experimental group (all *P*< 0.05).

**Conclusion:**

The foot skin protection technology demonstrates significant potential in enhancing foot skin condition.

## 1 Introduction

The population of elderly patients with diabetes in China is steadily rising alongside the aging demographic. Among the chronic complications affecting this demographic, diabetic foot ulcer (DFU) stands out as particularly prevalent, with peripheral neuropathy emerging as a significant independent risk factor for its development (1–3). Diabetic peripheral neuropathy (DPN) is a commonly encountered chronic complication in elderly patients with diabetes, characterized by neurological symptoms such as paresthesia, pain, and numbness (4). Roughly 30% to 40% of patients with diabetes, and up to 50% of those over 60 years old, experience peripheral neuropathy, which often leads to various foot skin problems and contributes significantly to the occurrence of DFU (5, 6). International guidelines emphasize the importance of regular assessment of foot skin condition and temperature to prevent DFU (7). Elderly individuals are more susceptible to skin damage compared to the general population. Given the chronic and insidious nature of DPN symptoms, early identification of foot skin problems in elderly patients is challenging but crucial for effective treatment and prognosis (8). Therefore, proactive nursing interventions aimed at early detection and management of skin problems in elderly patients with DPN are vital for preventing DFU.

Foot skin protection technology is a multidisciplinary collaboration involving departments such as endocrinology, dermatology, vascular surgery, orthopedics, specialized diabetic foot care clinics, and rehabilitation. This multidisciplinary team develops and applies the following specific techniques: (1) Biomechanics-based techniques to reduce foot pressure, such as pressure transfer techniques, callus trimming techniques, and the use of new dressings. (9) (2) Maintaining skin moisture, as diabetic peripheral neuropathy leads to issues like dry and cracked foot skin, which are risk factors for diabetic foot. For these issues, the care clinic and dermatology personnel conduct regular inspections and evaluations of patients and provide guidance on proper medication use. (3) Correction techniques for deformed toenails, involving trimming, filing, or reshaping the nails to make them more normal in shape, thereby reducing pressure and friction on surrounding tissues. (4) Individualized treatment, guidance, and medication for other emerging issues, including the three mentioned above (10). The skin problems are dynamically assessed, and intervention strategies are adjusted promptly. Ryder et al. (11) found that the occurrence of DFU is associated with autonomic nervous system damage. There is a close interaction between the autonomic nerves and skin keratinocytes and immune cells, playing an important regulatory role in skin physiological functions (12). Autonomic neuropathy can lead to reduced secretion of sweat glands and sebaceous glands, causing sweat dysfunction. When the skin moisture content is chronically low, haptens can more easily penetrate the skin, increasing the likelihood of sensitization. Additionally, reduced sweat can cause various skin issues such as dryness and scaling, compromising the skin’s integrity, damaging its barrier function, and increasing infection risk (13). Motor neuropathy can cause bone and joint damage and deformities, such as clawfoot deformity and Charcot joint deformity. The resulting dorsal and plantar flexion contractures create significant pressure points, altering the patient’s gait and leading to instability while walking. These changes can damage the skin on the feet, leading to ulcers. Motor neuropathy-induced muscle atrophy and deformity in the feet can continuously increase plantar pressure and cause abnormal pressure distribution, which are key factors in callus formation (14). Without timely intervention, persistent mechanical pressure may lead to DFU, and motor neuropathy can increase the risk of DFU in diabetic patients by up to 2.45 times (11). Sensory neuropathy causes positive symptoms such as tingling, burning, or pain, leading patients to scratch repeatedly. Concurrent ataxia or retinopathy increases the likelihood of falls. Negative signs mainly include the loss of protective sensation, with patients losing the ability to feel pain, pressure, and temperature in their feet, lacking self-protective awareness when injured, and being less likely to notice lesions. These abnormal sensations (either hypersensitivity or loss) can damage the skin on the feet, leading to ulcers (15). Currently, there are numerous protective measures for the foot skin of diabetic patients, but most interventions focus on single symptoms with limited consideration of disease-related factors. There are few targeted skin interventions for DPN, and there is a lack of comprehensive skin protection plans. Specific issues such as increased local skin tension due to motor neuropathy, hyperkeratosis caused by sensory neuropathy, and dryness and cracking resulting from impaired sweating function due to autonomic neuropathy are often not adequately addressed. The aim of this study is to evaluate and diagnose foot skin issues in elderly patients with DPN and explore the effectiveness of foot skin protection technology as an intervention method.

## 2 Participants and methods

### 2.1 Research participants

From June to August 2022, a total of 88 elderly patients with DPN were recruited from Shanghai Jiangsu Road Community Health Service Center and Xinhua Road Community Health Service Center. Inclusion criteria for participation were: (1) aged 60 years or older; (2) diagnosed with type 2 diabetes mellitus according to the 1999 WHO criteria; (3) diagnosed with diabetic peripheral neuropathy based on the 2013 Consensus on DPN Diagnosis and Treatment (16); (4) presence of one or more foot skin problems associated with DPN; (5) voluntary participation and signed the informed consent form. Exclusion criteria were: (1) presence of diabetic foot ulcers; (2) severe lower limb vascular problems related to diabetes mellitus; (3) history of amputation; (4) acute infections, severe musculoskeletal disorders, and so on; (5) unconsciousness, mental disorders, or non-cooperation with the research. Using a random number table, the participants were randomly assigned into two groups, with 44 patients in each group. However, 3 patients from each group were lost to follow-up, leaving a final cohort of 41 patients in the experimental group and 41 patients in the control group. Baseline data analysis revealed no significant differences between the two groups (all *P* > 0.05), ensuring comparability (see **Tables 1**–**3**). This study was registered in the Chinese Clinical Trials Registry with registration number ChiCTR2200066360 (detailed in **Appendix 1**). The study protocol, informed consent form, and relevant participant information were reviewed and approved by East China Hospital, with approval number 20220104 (detailed in **Appendix 2**), before commencement of the study.

**Table 1:** Comparison of the general data of the two groups.

Items	Category	Experimental group (*n* = 44)	Control group (*n *= 44)	*t*/*χ* ^2^/Z	*P*
Sex [n (%)]	Male	25(56.82)	28(63.64)	0.427	0.513
	Female	19(43.18)	16(36.36)		
Age [n (%)]	60-69	13(29.55)	16(36.36)	0.398	0.530
	70-79	22(50.00)	20(45.45)		
	≥80	9(20.45)	8(18.19)		
BMI [M(P_25_,P_75_),kg/m^2^]		24.39 (22.71,26.3)	23.65 (22.15,25.31)	-0.822	0.411
Education degree [n (%)]	Elementary school and below	3(6.82)	3(6.82)	5.239	0.155
	Junior school	30(68.18)	20(45.45)		
	High school/Technical secondary school	7(15.91)	12(27.27)		
	Junior college or above	4(9.09)	9(20.45)		
Living condition [n (%)]	Living with family	11(25)	10(22.73)	0.063	0.803
	Living alone	33(75)	34(77.27)		
Family income (in RMB)	<2000	1(2.27)	1(2.27)	1.298	0.73
	2000-5000	20(45.45)	15(34.09)		
	5000-8000	20(45.45)	22(50)		
Family income (in RMB)	>8000	3(6.82)	5(11.36)		
Diabetes disease course ( $\bar{x}\pm s$ , year)		14.98 ± 10.21	12.91 ± 8.36	-0.737	0.461
Fasting blood glucose [M (P_25_, P_75_), mmol]		7.55(6.50,8.85)	7.50(6.73,7.88)	-0.38	0.704
Random blood glucose [M (P_25_, P_75_), mmol]		10.65(9.20,10.98)	9.91(8.02,10.8)	-1.659	0.097
Glycosylated hemoglobin [M (P_25_, P_75_), %]		8.27(7.30,9.1)	8.06(7.93,8.06)	-1.374	0.17
Treatment [n (%)]	No medication	14(31.82)	10(22.73)	0.917	0.338
	Oral medication	31(70.45)	32(72.73)	0.056	0.813
	Insulin	4(9.09)	6(13.64)	0.451	0.502
Complications of diabetes					
Peripheral vascular lesions [n (%)]	No	27(61.36)	34(77.27)	2.618	0.106
	Yes	17(38.64)	10(22.73)		
Diabetic nephropathy [n (%)]	No	35(79.55)	37(84.09)	0.306	0.58
	Yes	9(20.45)	7(15.91)		
Retinopathy [n (%)]	No	33(75)	35(79.55)	0.259	0.611
	Yes	11(25)	9(20.45)		
Comorbidity [n (%)]	0–1 type of disease	13(29.55)	14(31.82)	5.904	0.206
	2 types of diseases	11(25)	11(25)		
	3 types of diseases	11(25)	10(22.73)		
	>3 types of diseases	5(11.36)	9(20.45)		
Smoking history [n (%)]	No	27(61.36)	33(75)	1.886	0.17
	Yes	17(38.64)	11(25)		
Drinking history [n (%)]	No	30(68.18)	36(81.82)	2.182	0.14
	Yes	14(31.82)	8(18.18)		
Self-care ability of daily living [n (%)]	No dependency	37(84.09)	41(93.18)	1.805	0.179
	Mild dependency	7(15.91)	3(6.82)		

**Table 2:** Comparison of foot skin problems between the two groups.

Items	Category	Experimental group (*n* = 44)	Control group (*n *= 44)	*t*/*χ* ^2^/Z	*P*
Dryness [n (%)]	No	4(9.09)	9(20.45)	2.256	0.133
	Yes	40(90.91)	35(79.55)		
Degree of dryness [M (P_25_, P_75_), scores]		2(2,3)	2(1,3)	-1.699	0.089
Desquamation [n (%)]	No	13(29.55)	14(31.82)	0.053	0.817
	Yes	31(70.45)	30(68.18)		
Cracking [n (%)]	No	27(61.36)	32(72.73)	1.286	0.257
	Yes	17(38.64)	12(27.27)		
Eczema [n (%)]	No	34(77.27)	40(90.91)	3.058	0.08
	Yes	10(22.73)	4(9.09)		
Calluses [n (%)]	No	12(27.27)	20(45.45)	3.143	0.076
	Yes	32(72.73)	24(54.55)		
Corns [n (%)]	No	39(88.64)	43(97.73)	1.61	0.205
	Yes	5(11.36)	1(2.27)		
Bullae [n (%)]	No	43(97.73)	44(100)	0	1
	Yes	1(2.27)	0(0)		
Fungal infection [n (%)]	No	17(38.64)	25(56.82)	2.915	0.088
	Yes	27(61.36)	19(43.18)		
Edema [n (%)]	No	38(86.36)	39(88.64)	2.890	0.089
	Yes	6(13.64)	5(11.36)		
Itching [n (%)]	No	13(29.55)	21(47.73)	3.068	0.08
	Yes	31(70.45)	23(52.27)		
Itching degree [M (P_25_, P_75_), scores]		4.5(0,6.75)	2.5(0,6)	-1.594	0.111
Numbness [n (%)]	No	36(81.82)	39(88.64)	0.812	0.367
	Yes	8(18.18)	5(11.36)		
Sensory loss [n (%)]	No	16(36.36)	21(47.73)	1.166	0.28
	Yes	28(63.4)	23(52.27)		
Acmesthesia [n (%)]	No	40(90.91)	43(97.73)	0.848	0.357
	Yes	4(9.09)	1(2.27)		
Pain [n (%)]	No	38(86.36)	42(95.45)	1.238	0.266
	Yes	6(13.64)	2(4.55)		
Formication [n (%)]	No	44(100)	42(95.45)	0.512	0.474
	Yes	0(0)	2(4.55)		
Pigmentation [n (%)]	No	27(61.36)	32(72.73)	1.286	0.257
	Yes	17(38.64)	12(27.27)		
Paleness [n (%)]	No	41(93.18)	40(90.91)	0	1
	Yes	3(6.82)	4(9.09)		
Decreased temperature [n (%)]	No	30(68.18)	24(54.55)	1.725	0.189
	Yes	14(31.82)	20(45.45)		
Increased temperature [n (%)]	No	37(84.09)	42(95.45)	3.094	0.079
	Yes	7(15.91)	2(4.55)		
Tinea pedis scores [M (P25, P75), scores]		3 (2,3)	3 (2,3)	-0.029	0.977

**Table 3:** Comparison of plantar callus assessment data between the two groups ( $\bar{x}$ ± s ).

Items	Experimental group (*n* = 44)	Control group (*n *= 44)	*t*	*P*
Area ( $\bar{x}\pm s$ , cm^2^)	4.2 ± 0.70	4.26 ± 0.78	-0.216	0.829
Thickness ( $\bar{x}\pm s$ , mm)	1.49 ± 0.85	1.44 ± 0.69	-0.077	0.938
Walking comfort $\left(\bar{x}\pm s\right)$	3.25 ± 0.57	2.45 ± 0.40	-1.850	0.064
Resting comfort $\left(\bar{x}\pm s\right)$	3.8 ± 0.9	3.25 ± 0.61	-2.638	0.080

The random grouping was conducted using SPSS software with the following steps: (1) Study subjects were assigned numbers; (2) A fixed starting value of 20220608 was set in the random number generator; (3) A random number sequence was generated by computing variables; (4) The generated random numbers were randomly divided into two groups in a 1:1 ratio and labeled as groups “1” and “2”; (5) The SPSS-generated random numbers and their corresponding group assignments were noted on cards, which were then placed in opaque, sealed envelopes, and the envelopes were labeled with serial numbers. The envelopes were handed to another research team member who was unaware of the grouping details. This member distributed the envelopes according to the order in which the subjects were included in the study. After the baseline measurements, the envelopes were opened on the spot. Patients with cards marked “1” received the foot skin protection plan based on elderly DPN patients, while those with cards marked “2” received the standard community foot skin care guidance for elderly DPN patients.

The flow diagram of the study process is shown in **Supplementary Figure S1**.

### 2.2 Methods

#### 2.2.1 Development of foot skin protection technology in elderly patients with DPN

Following the principles of evidence-based medicine and drawing from previous clinical studies, alongside insights from real-world clinical practice, the foot skin protection technology tailored for elderly patients with DPN was developed. This technology encompasses various strategies, including biomechanics-based plantar decompression techniques, foot callus treatment methods, and management techniques for diabetic bullae (17).

#### 2.2.2 Intervention methods

Patients in both the control and experimental groups received diabetic medication, nursing care, and health education regarding diabetic foot management. In the control group, patients attended community outpatient clinics where their concerns were addressed by outpatient specialist nurses. Conversely, patients in the experimental group were provided with the foot skin protection technology specifically tailored for elderly patients with DPN, developed by the podiatry clinic of East China Hospital. They were instructed to attend follow-up visits every 2 weeks at the integrated podiatry clinic of East China Hospital, with specific follow-up times determined accordingly. After a 3-month intervention period, the patients’ adherence to the skin protection plan, foot care practices, occurrence of foot problems, and the efficacy of the intervention were monitored and assessed during follow-up visits.

The standard community nursing guidance provided to the control group was as follows:

(1) Health Education: Conducted through foot care knowledge lectures and distribution of health education guidebooks. This included daily foot skin inspections, daily care routines, exercise precautions, proper selection of shoes and socks, toenail trimming prevention, and daily prohibitions for peripheral neuropathy.
(2) Clinic Visits for Foot Problems: When patients encountered foot problems, they visited the foot clinic where specialized nurses addressed their issues. The main content included assessing changes in foot skin, such as dryness, calluses, infections, damage, and abnormal sensations. Individualized treatment and health guidance were provided for existing skin problems.
(3) Monthly Telephone Follow-Ups: Conducted once a month, each call lasting 10–15 minutes. The primary focus was to understand any abnormal issues with the patient’s foot skin and provide guidance over the phone for any skin problems that occurred.

The foot skin protection technology provided to the experimental group was as follows:

(1) Individualized Education and Professional Support: Based on the overall and foot skin care assessment results, patients were provided with personalized education and professional technical support. Specific content included daily foot skin inspection, daily care, exercise precautions, proper selection of shoes and socks, standard toenail trimming, and daily prohibitions for peripheral neuropathy. A health education guidebook was distributed.
(2) Patient Involvement in Decision-Making: Patients were encouraged to actively participate in the decision-making process of the care intervention plan, enhancing their sense of health responsibility. This included daily skin care plans, exercise plans, and dietary aspects.
(3) Knowledge Lectures: Knowledge lectures were conducted in the 1st, 5th, and 9th weeks, focusing on the dangers of diabetic foot complications and how to prevent them, foot cleaning and moisturizing, proper toenail trimming, and selection and wearing of shoes and socks. Each lecture lasted 30 minutes. During the lectures, on-site interactions with patients were conducted, including a Q&A session to help patients recognize the importance of timely addressing foot skin problems and improve their awareness of foot protection.
(4) Emotional Support: Emotional support was provided through face-to-face and telephone communication and establishing a WeChat group to stay in contact with patients, listen to their feelings, and provide timely feedback. For elderly patients living alone, communication frequency was increased as needed. Monthly psychological counseling sessions were provided, offering emotional support. Three additional counseling sessions, each lasting 30 minutes, were scheduled in the 2nd, 7th, and 11th weeks. The themes were “The Impact of Diabetic Foot Skin Issues on Life and Emotional Reactions,” “Positive Ways to Cope with Foot Complications,” and “Emotional Management and Self-Regulation Techniques.”
(5) Follow-Up Appointments: Patients were guided to visit the Integrated Foot Clinic at Huadong Hospital every two weeks for a follow-up. Specific follow-up times were determined. During these visits, the execution of the patient’s skin protection plan, foot care behaviors, occurrence of foot problems, and the effectiveness of the application were tracked and evaluated.
(6) Adjustment of Intervention Plan: The content of the skin intervention care plan was adjusted promptly, and patients’ questions were answered.

To keep consistency during the implementation process, during the intervention period, participants regularly received clinical monitoring and guidance, including assessments of foot skin condition, monitoring of intervention effectiveness, and training in foot care techniques. This ensured participants correctly understood and executed the intervention plan, with timely adjustments made to the interventions to suit individual situations.

#### 2.2.3 Quality control measures

Drawing from evidence-based medicine principles and prior clinical research findings, the foot skin protection technology for elderly patients with DPN was developed, and was meticulously refined through expert consultation to ensure its scientific rigor and safety. To mitigate selective bias, the randomization scheme was concealed. Standardized assessment and intervention protocols were established through unified training for all participating medical personnel. Regular follow-up visits were conducted biweekly to monitor progress. Additionally, a monthly 30-minute knowledge lecture covering diabetic foot complications, preventive measures, foot hygiene practices, proper toenail trimming techniques, footwear selection, and usage precautions was organized. During these sessions, interactive discussions were facilitated to engage patients directly, fostering an environment for queries and clarifications, thereby enhancing patients’ awareness of foot protection and the importance of timely treatment for foot skin issues.

#### 2.2.4 Effect evaluation

##### 2.2.4.1 Process indicators

Foot care behavior: The Foot Skin Self-Management Behavior Questionnaire consists of 8 items: “Daily skin inspection,” “Daily skin cleaning,” “Use of mild bath soap,” “Drying skin with a soft towel,” “Daily application of moisturizer,” “Wearing soft and breathable socks,” “Taking measures when exposed to sunlight,” and “Adhering to doctor’s guidance.” Scoring is based on a Likert 5-point scale, with scores ranging from 0–4 for each item, totaling a maximum of 20 points. Higher scores indicate better foot skin care by the patient.

Health-promoting behavior: This scale is based on the Health Promoting Lifestyle Profile (HPLP) developed and revised by Walker et al. (18) The scale uses a Likert 4-point scoring method (Never, Sometimes, Usually, Always). The overall Cronbach’s α coefficient for the scale is 0.94, with Cronbach’s α coefficients for the six dimensions ranging from 0.79 to 0.87. The Cronbach’s α coefficient for this dimension is 0.87.

##### 2.2.4.2 Outcome indicators

Incidence and severity of foot skin problems: Common clinical measurement tools were used, including the Overall Dry Skin Score (19), Visual Analog Scale (20), Total Symptom Score (21) for athlete’s foot symptoms and signs, and the Diabetic Foot Comfort Assessment Plate (22).

Service Satisfaction (Client Satisfaction Tool): This scale is a patient satisfaction survey designed by Bear et al., using the Cox Health Behavior Interaction Model as a framework. This study uses the translated version by Bai Yating. The Cronbach’s α value and test-retest reliability of this scale are 0.938 and 0.974, respectively. The Pearson correlation coefficients for each item range from 0.630 to 0.919, and the Item-Level Content Validity Index for each item is 1.

#### 2.2.5 Data collection

The data collection procedures were as follows: (1) Secured support for the study from the hospital and two community health service centers. (2) Community general practitioners were responsible for recruiting subjects who met the inclusion and exclusion criteria. Researchers provided detailed explanations and training on these criteria. (3) Researchers provided informed consent forms to the study subjects. These forms had been approved by the ethics committee and included detailed explanations of the study’s purpose, process, voluntariness, confidentiality, and harmlessness. The study commenced after the subjects signed the informed consent forms. (4) Before the study began, researchers collected general and disease-related information from the patients. After collecting the data, research members carefully checked and verified the information. If there were any missing items, they promptly inquired about the reasons and filled in the missing information; otherwise, the sample was considered invalid, ensuring the data was qualified. (5) Researchers assessed the patients’ foot skin problems, health behaviors, and service satisfaction twice: before the intervention began and three months after the intervention.

#### 2.2.6 Statistic analysis

Statistical analysis was performed using SPSS 25.0 software.

To calculate the sample size, if estimating sample size using Pearson’s Chi-squared test, the formular is $n_{1}=n_{2}=\frac{{\left[\mu_{a/2}\sqrt{2\bar{p}\left(1-\bar{p}\right)}+\mu_{\beta }\sqrt{p_{1}\left(1-p_{1}\right)+p_{2}\left(1-p_{2}\right)}\right]}^{2}}{{\left(p_{1}-p_{2}\right)}^{2}}$ . If Fisher’s exact test or a corrected Chi-squared test for continuity was used, the formular is $n^{\prime }=\frac{n}{4}{\left(1+\sqrt{1+\frac{4}{n\left|p_{1}-p_{2}\right|}}\right)}^{2}$ , in which α and β represent the probabilities of type I and type II errors, conventionally set at: two-sided α = 0.05, β = 0.1, Uα/2 = 1.96, Uβ = 1.282. Referring to the study by Yang et al. (23), the index of abnormal skin sensation changed from P1 = 0.51 to P2 = 0.15 with intervention, applying the formula yields: for Pearson’s Chi-squared test, each group requires 35 cases, adjusted for 10% loss to follow-up, totaling 78 cases with 39 per group; for Fisher’s exact test or corrected Chi-squared test for continuity, each group requires 38 cases, adjusted for 10% loss to follow-up, totaling 88 cases with 44 per group.

Continuous variables following a normal distribution are presented as mean ± standard deviation, while those not conforming to normal distribution are described as median and quartile. Categorical data are expressed as frequency and percentage. For normally distributed continuous data, independent samples *t*-test was employed to compare between the two groups. For non-normally distributed continuous data or ordinal categorical data, the Mann–Whitney U test, a non-parametric test, was utilized. The chi-squared test was applied to compare differences between the two groups for categorical data. Continuity correction in the chi-squared test was implemented when the theoretical frequency was ≥ 1 and< 5, and the Fisher’s exact probability method was used when the theoretical frequency was< 1. Paired sample *t*-test was utilized to compare pre- and post-intervention data within each group for normally distributed continuous variables, while the Wilcoxon test, a paired non-parametric test, was used in other cases. A significance level of *P*< 0.05 was considered statistically significant.

## 3 Results

### 3.1 Comparison of foot skin problems between the two groups

After three months of intervention, the experimental group exhibited lower incidences of various foot skin problems compared to the control group. Specifically, the incidence rates in the experimental group for skin dryness, desquamation, skin cracking, eczema, fungal infection, and itching were 34.15% (14 cases), 21.95% (9 cases), 14.63% (6 cases), 2.44% (1 case), 12.2% (5 cases), and 17.07% (7 cases), respectively. In contrast, the control group had higher incidence rates for these issues: 60.98% (25 cases), 48.78% (20 cases), 26.83% (11 cases), 14.63% (6 cases), 34.15% (14 cases), and 41.46% (17 cases), respectively. These differences were statistically significant (χ2 = 5.917, 6.455, 6.212, 9.390, 5.549, 5.891; *P* = 0.015, 0.011, 0.013, 0.026, 0.018, 0.015) (See **Table 4** for details).

**Table 4:** Comparison of foot skin problems between the two groups after intervention.

Items	Before/After intervention	Experimental group (*n* = 44)	Control group (*n *= 44)	*t*/*χ* ^2^/Z	*P*
Dryness [n (%)]	Before intervention	40(90.91)	35(79.55)	2.256	0.133
	After intervention	14 (34.15)	25 (60.98)	5.917	0.015
Degree of dryness [M (P_25_, P_75_), scores]	Before intervention	2(2,3)	2(1,3)	-1.699	0.089
	After intervention	1(0,2)	1(1,2)	-2.097	0.036
Desquamation [n (%)]	Before intervention	31(70.45)	30(68.18)	0.053	0.817
	After intervention	9 (21.95)	20 (48.78)	6.455	0.011
Cracking [n (%)]	Before intervention	17(38.64)	12(27.27)	1.286	0.257
	After intervention	6 (14.63)	11(26.83)	6.212	0.013
Eczema [n (%)]	Before intervention	10(22.73)	8(18.18)	3.058	0.08
	After intervention	1(2.44)	6(14.63)	9.390	0.026
Corns [n (%)]	Before intervention	5(11.36)	1(2.27)	1.61	0.205
	After intervention	2 (4.88)	1(2.44.)	0	0.95
Bullae [n (%)]	Before intervention	1(2.27)	0(0)	1.011	1
	After intervention	1 (2.44)	0 (0.00)	1.012	0.314
Fungal infection [n (%)]	Before intervention	27(61.36)	19(43.18)	2.915	0.088
	After intervention	5 (12.20)	14 (34.15)	5.549	0.018
Edema [n (%)]	Before intervention	6(13.63)	5(11.36)	2.890	0.089
	After intervention	1 (2.44)	2 (4.88)	0.346	0.556
Itching [n (%)]	Before intervention	31(70.45)	23(52.27)	3.068	0.08
	After intervention	7 (17.07)	17 (41.46)	5.891	0.015
Itching assessment [M (P_25_, P_75_), scores]	Before intervention	4.5(0-6.75)	2.5(0-6)	-1.594	0.111
	After intervention	1(0, 2.)	2(0, 5)	-2.695	0.007
Sensory loss [n (%)]	Before intervention	8(18.18)	5(11.36)	0.812	0.367
	After intervention	6 (0.00)	4 (9.76)	4.205	0.312
Acmesthesia [n (%)]	Before intervention	4(9.09)	1(2.27)	0.848	0.357
	After intervention	4 (9.76)	1 (2.44)	1.917	0.166
Pain [n (%)]	Before intervention	6(13.64)	2(4.55)	1.238	0.266
	After intervention	5 (12.20)	2 (4.88)	1.406	0.236
Formication [n (%)]	Before intervention	0(0)	2(4.55)	0.512	0.474
	After intervention	1(0)	2(4.88)	0	1
Pigmentation [n (%)]	Before intervention	17(38.64)	15(34.09)	1.286	0.257
	After intervention	16 (39.02)	15(34.15)	0.225	0.655
Paleness [n (%)]	Before intervention	3(6.82)	4(9.09)	0	1
	After intervention	2 (4.88)	4 (9.76)	0.719	0.396
Decreased temperature [n (%)]	Before intervention	14(31.82)	20(45.45)	1.725	0.189
	After intervention	8 (19.51)	16 (39.02)	3.77	0.052
Increased temperature [n (%)]	Before intervention	7(15.91)	2(4.55)	3.094	0.079
	After intervention	3 (7.32)	2 (4.88)	0.213	0.644
Tinea pedis scores [M (P25, P75), scores]	Before intervention	3(2,3)	3(2,3)	-0.029	0.977
	After intervention	1(0,1)	2(0,2)	-2.924	0.003

### 3.2 Comparison of the severity of foot skin problems between the two groups

Based on statistical analysis, it was found that the area, thickness, and comfort levels of plantar calluses in the experimental group were significantly better than those in the control group (*t* =-2.486, -2.816, -2.693, -2.875; *P* = 0.013, 0.005, 0.007, 0.004) (**Table 5**). Additionally, the scores for dryness, itching, and tinea pedis in the experimental group were significantly lower compared to the control group (Z = -2.097, -2.695, -2.924; *P* = 0.036, 0.007, and 0.003, respectively) (**Table 4**).

**Table 5:** Comparison of plantar callus assessment between the two groups after intervention ( $\bar{x}$ ± s ).

Items	Before/After intervention	Experimental group (*n* = 44)	Control group (*n *= 44)	*t*	*P*
Area ( $\bar{x}\pm s$ , cm^2^)	Before intervention	4.2 ± 0.70	4.26 ± 0.78	-0.216	0.829
	After intervention	3.80 ± 0.73	4.04 ± 0.66	-2.486	0.013
Thickness ( $\bar{x}\pm s$ , mm)	Before intervention	1.49 ± 0.85	1.44 ± 0.69	-0.077	0.938
	After intervention	1.02 ± 0.55	1.44 ± 0.81	-2.816	0.005
Walking comfort $\left(\bar{x}\pm s\right)$	Before intervention	3.25 ± 0.57	2.45 ± 0.4	-1.850	0.064
	After intervention	1.58 ± 0.49	2.42 ± 0.63	-2.693	0.007
Resting comfort $\left(\bar{x}\pm s\right)$	Before intervention	3.8 ± 0.9	3.25 ± 0.61	-2.638	0.080
	After intervention	1.0 ± 0.45	1.64 ± 0.39	-2.875	0.004

## 4 Discussion

### 4.1 Diabetic peripheral neuropathy can cause a variety of skin problems

Ryder et al. (24) discovered a significant interplay between autonomic nerves, skin keratinocytes, and immune cells, which holds pivotal importance in regulating the physiological functions of skin (12). Autonomic neuropathy can trigger reduced secretion from sweat and sebaceous glands, resulting in dysfunction in perspiration, dryness, scaling, and various other skin issues (9). This can disrupt skin integrity, compromise barrier function, and heighten the risk of infections (13). Motor neuropathy-induced muscle atrophy and foot deformities can elevate plantar pressure and cause abnormal pressure distribution, crucial factors contributing to callus formation (14). Sensory neuropathy primarily manifests as a loss of protective sensation, rendering patients unable to perceive pain, pressure, or temperature changes in their feet. Consequently, patients may lack awareness of injuries and struggle to detect lesions, which can predispose them to foot skin damage (25).

### 4.2 Foot skin protection technology can reduce the occurrence of foot skin problems in elderly patients with DPN

Skin issues like dryness, cracking, eczema, itching, and fungal infections are common precursors to ulcers in diabetic patients, necessitating timely identification and treatment to prevent foot skin damage and subsequent infection (26). This study’s findings revealed a significant reduction in the incidence of these foot skin problems, including dryness, scaling, cracking, eczema, itching, and fungal infections, after a three-month intervention in the experimental group compared to the control group (*P*< 0.05). This suggests that foot skin protection technology can effectively lower the occurrence of such issues. Elderly patients with DPN often experience foot paresthesia, making it difficult for them to detect foot skin damage promptly, thus increasing their susceptibility to skin damage (24). Consequently, early detection and treatment of foot skin issues represent effective measures to prevent the onset and progression of foot ulcers (27).

### 4.3 Foot skin protection technology can reduce the severity of foot skin problems in elderly patients with DPN

Foot calluses can greatly impact a patient’s comfort, especially during walking, leading to a sensation of discomfort akin to having a foreign object present (28). These uncomfortable sensations can evoke negative emotions like anxiety and depression, fostering a sense of helplessness that hinders the adoption of healthy behavior. Following a three-month intervention, the experimental group exhibited significantly lower comfort scores associated with foot calluses compared to the control group (*P*< 0.05). It is important to note that the comfort score is inversely proportional to the level of comfort, with a lower score signifying an enhanced level of comfort. Employing evidence-based methods for timely and standardized treatment of foot calluses can mitigate the risk of skin damage and infection. Callus excision proves effective in relieving pressure on the plantar surface for patients. Research by Xu et al. (29) demonstrated that prompt callus treatment in 60 patients with diabetes effectively curbed the incidence of foot ulcers, enhanced quality of life, and reduced anxiety levels. Additionally, scores for foot skin dryness, itching, and athlete’s foot were markedly diminished in the experimental group compared to the control group (*P*< 0.05), indicating that foot skin protection technology not only reduces the occurrence of foot skin issues but also alleviates dryness, itching, and symptoms of athlete’s foot. Through standardized interventions, effective maintenance of skin moisture and oil secretion can be achieved, reducing the occurrence of skin problems related to dryness. Customized interventions based on individual and environmental characteristics are designed to meet specific needs. These interventions include guiding the elderly on proper foot inspection, cleaning, selection of suitable shoes and socks, nail trimming, appropriate exercise, choosing suitable moisturizing products, using proper pressure-relief footwear/assistive devices, and following medical advice. This approach helps patients understand health-related information and emphasizes the importance of foot skin care. By employing engaging methods such as “on-site demonstrations,” “hands-on teaching,” “on-site assessments,” and “prize quizzes,” elderly patients or their caregivers are taught essential foot care knowledge and skills. For issues that patients cannot resolve on their own, timely and appropriate handling and protection are provided. Patients are informed about when and how to seek medical attention, ensuring that they receive prompt and proper treatment for foot problems.

There are some limitations in this study. First, due to time constraints, the long-term care outcomes of patients were not discussed, and the long-term effects of interventions cannot be determined. Second, this study included subjects from only two community hospitals, thus the representativeness of the study is limited. Future research should be conducted in different regions and hospitals of various levels to broaden the sample sources, increase the overall sample size, and enhance the representativeness of the research results. In addition, the follow-up period should be expanded to continuously track the intervention effects of foot skin protection in elderly DPN patients and analyze the long-term impact of the protective measures on patients.

## 5 Conclusion

In this study, we elucidated the extent of foot skin issues among patients with DPN and investigated the efficacy of foot skin protection technology. Through the comparison of results from the experimental and control groups, it is evident that the utilization of this technology markedly diminishes both the occurrence and severity of skin issues, leading to improved patient comfort and overall well-being. This study offers a crucial theoretical and practical foundation for nursing care in elderly patients with diabetes. Future research should delve into the operational complexities and long-term impacts of foot skin protection technology, alongside its dissemination and implementation in clinical settings, to furnish more efficacious strategies for caring for elderly patients with diabetes.

## Data Availability

The original contributions presented in the study are included in the article/**Supplementary Material**. Further inquiries can be directed to the corresponding authors.

## Supplementary material

Supplementary Figure 1 A flow diagram of study process.
