# Supplementary figures and images for: Effectiveness of foot skin protection technology in elderly patients with diabetic peripheral neuropathy
Source: Front Endocrinol (Lausanne). 2024 Aug 19;15:1411657. doi: 10.3389/fendo.2024.1411657 (PMC11367785; doi:10.3389/fendo.2024.1411657)

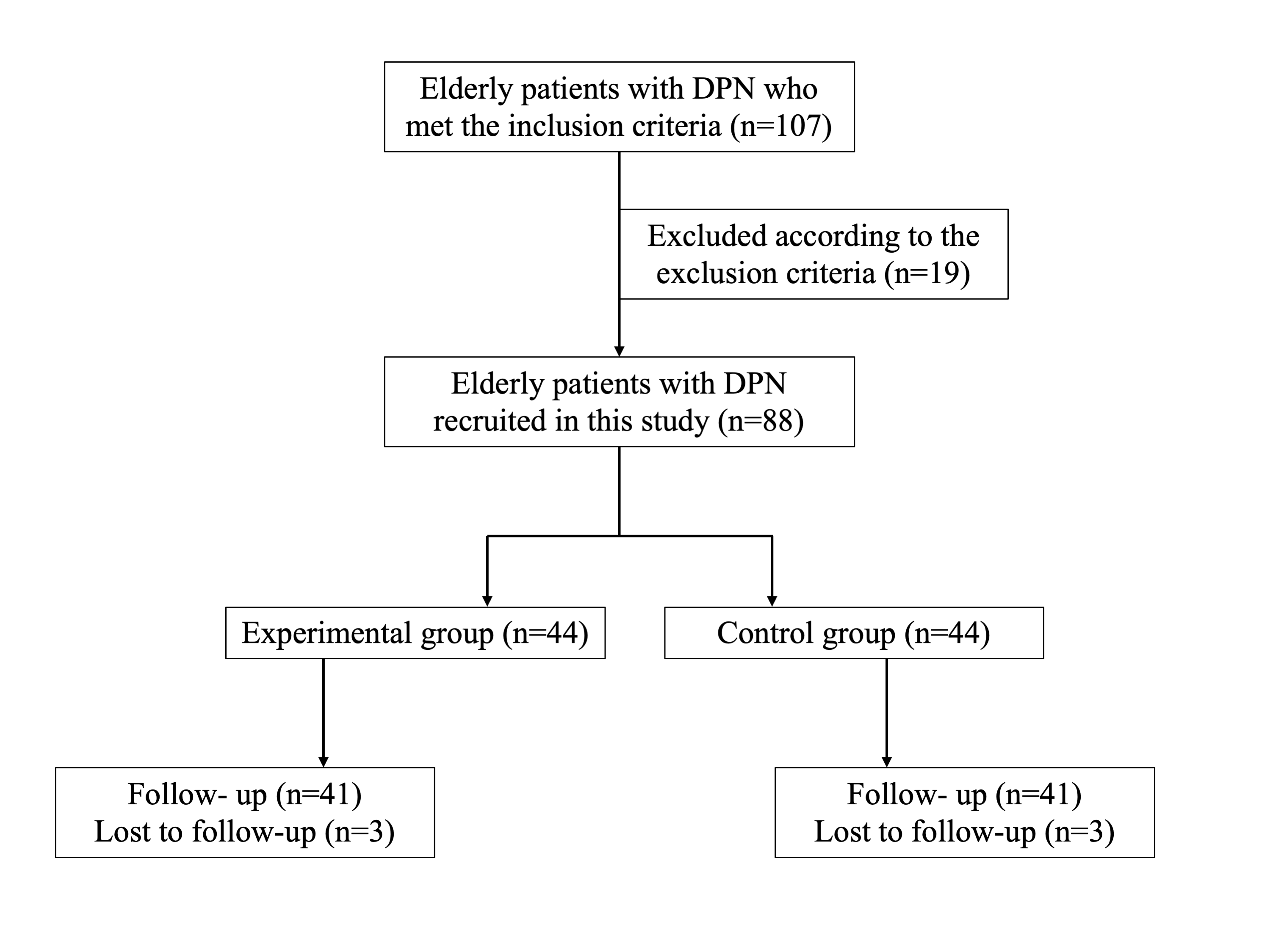

Supplement: Supplementary Figure 1 — A flow diagram of study process. [file Image1.tiff]
